# Milk component, farm, and industry-level factors associated with elevated free fatty acids in bulk tank milk on conventional dairy farms

**DOI:** 10.3168/jdsc.2025-0805

**Published:** 2025-08-20

**Authors:** Hannah M. Woodhouse, Stephen J. LeBlanc, Trevor J. DeVries, Karen J. Hand, David F. Kelton

**Affiliations:** 1 Department of Population Medicine, University of Guelph, Guelph, ON, Canada N1G2W1; 2 Department of Animal Biosciences, University of Guelph, Guelph, ON, Canada N1G2W1; 3 Precision Strategic Solutions, Puslinch, ON, Canada N0B2J0

## Abstract

Elevated concentrations of free fatty acids (FFA) in bulk tank milk indicate excessive milk fat breakdown. Levels exceeding the sensory threshold of ≥1.20 mmol FFA/100 g of milk fat compromise milk quality. Average FFA levels on Ontario (ON), Canada, conventional (CON) dairy farms are highest in the early fall months, and this study aimed to identify the factors associated with elevated FFA. A subset of milk-recorded ON CON dairy farm bulk tanks (n = 3,009) with FFA data between August 2018 and December 2022 were analyzed in a cross-sectional study. A mixed-effects multivariable logistic regression model was constructed with elevated monthly mean FFA (≥1.2 mmol FFA/100 g of fat) as the outcome. Monthly average milk components, the number of incentive days (a feature of the Canadian supply management system that allows producers to ship more milk when the domestic demand exists), and milking system were fixed effects in the model, and bulk tank was a random effect. The final dataset included 148,965 observations from 3,009 bulk tanks over 53 mo. The average monthly bulk tank FFA was 0.83 mmol/100 g of fat, and 7% (n = 10,016) of monthly average bulk tank FFA concentrations were elevated. Fifty-three percent of samples came from tiestall farms (n = 1,578), 29% from parlor-milked herds (n = 878), and 18% from farms with automated milking systems (AMS; n = 553). The final reduced model indicated that the odds of monthly average elevated FFA were higher for tiestall farms (odds ratios [OR] = 28.6) and AMS farms (OR = 10.9) when compared with parlor farms. Tiestall farms had higher odds of monthly average elevated FFA than AMS farms (OR = 2.6). The odds of monthly average elevated FFA were greater during months with 3 incentive days compared with none (OR = 1.6). Lower milk protein content was associated with increased odds of monthly average elevated FFA (OR = 33.2 per weighted volume percentage of milk protein decrease), and higher BactoScan (BSN) was associated with increased odds of monthly average elevated FFA (OR = 3.0 per unit increase in BSN (1,000 bacteria per mL more). The results demonstrated that nonparlor milking systems, months with 3 production incentive days, lower milk protein levels, and higher BSN were associated with elevated monthly average FFA levels in ON CON dairy herds.

## Highlights

• Milk analyses were conducted on Ontario milk-recorded conventional dairy farms.
• Average milk FFA levels were compared with farm characteristics.
• The average FFA was 0.83 mmol/100 g of fat, and 7% of samples were elevated.
• Nonparlor milking systems and incentive days were associated with elevated FFA.
• Low milk protein and high milk bacteria were associated with elevated FFA.

## Graphical Abstract

**Figure:**
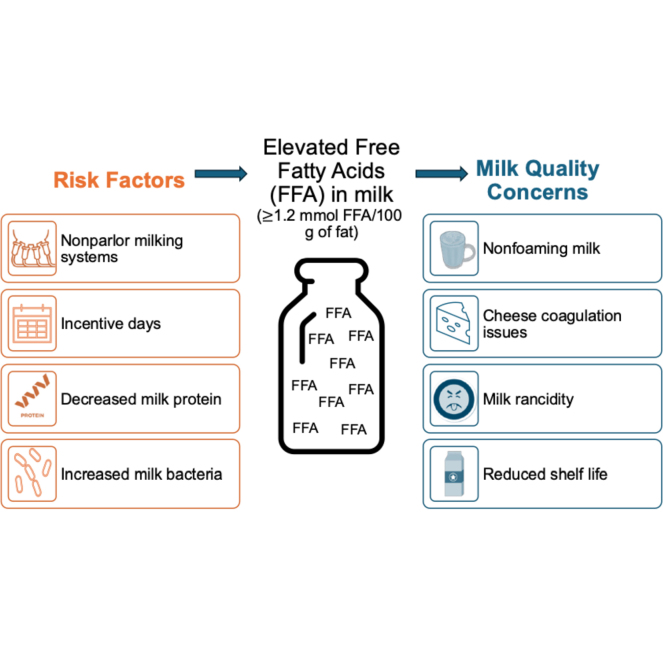
**Summary:** Elevated levels of free fatty acids (FFA) are a concern for milk quality. A subset of Ontario, Canada, dairy farms was analyzed, and a mixed-effects logistic regression model was used to compare milk component, farm, and industry factors with the average monthly FFA. The average monthly FFA concentration was 0.83 mmol/100 g of fat, with 7% of averages having an elevated FFA concentration (≥1.2 mmol/100 g of fat). Results showed significant associations between elevated FFA and nonparlor milking systems, months with 3 incentive days (a feature of the Canadian supply management system that allows producers to ship more milk when the domestic demand exists), lower milk protein, and increased milk bacteria.

Free fatty acids (**FFA**) are natural components of milk but at low levels (Hanuš et al., 2008). At concentrations ≥1.20 mmol/100 g of fat (Wiking et al., 2017), milk quality concerns arise, including milk nonfoaming, rancidity, reduced shelf life, and cheese coagulation issues (Deeth, 2006; Skeie, 2007; Kamath et al., 2008).

Free fatty acids are products of milk fat breakdown, referred to as lipolysis (Lindmark Månsson, 2008). They result from triacylglycerol (**TAG**) dissociation when the milk fat globule membrane is disrupted and TAG molecules contained inside are exposed to lipolytic enzymes (Deeth, 2006). Some of the lipolytic enzymes, such as lipoprotein lipase, naturally occur in milk (referred to as spontaneous lipolysis), whereas others come from bacteria (referred to as bacterial lipolysis; Deeth, 2006). Induced lipolysis is another mechanism for FFA production and occurs when there is physical stress on the milk (Deeth, 2006).

Since August 2018, the milk marketing board of Ontario (**ON**), Canada (Dairy Farmers of Ontario; **DFO**), has tested bulk tank milk FFA levels on all licensed dairy farms at each pick-up (usually every other day). Free fatty acid levels are reported to producers as a milk quality measure along with other routine milk composition data, including fat, protein, BactoScan (**BSN**) by FOSS, and SCC.

Bactoscan and SCC are also measures of milk quality and may be related to FFA. Elevated BSN could increase bacterial lipolysis to yield more FFA (Reguillo et al., 2018). Somatic cell count represents the number of white blood cells in milk, and researchers have demonstrated an association between higher SCC and higher FFA due to increased concentrations of lipoprotein lipase in high SCC milk (Barbano et al., 2006; Chipilev et al., 2017). Smaller herds have been associated with increased bulk tank SCC and FFA compared with larger herds due to one cow's susceptible milk representing a more significant proportion of the bulk tank (Oleggini et al., 2001; Mikulová, 2011).

Like BSN and SCC, FFA has a seasonal pattern (Woodhouse et al., 2025b). On average, the highest FFA levels occur in the fall months and the lowest occur in the spring months (Woodhouse et al., 2025b). During the fall months, there is an increase in milk demand (due to upcoming holidays such as Thanksgiving and Christmas; DFO, 2010; Shock et al., 2015). The industry responds by adding incentive days, which is a feature of the Canadian supply management systems that allows dairy producers to ship milk beyond their quota allotment (a permitted amount of milk production for each producer based on milk fat [kg/d]).

Incentive days are assigned by the month, with a maximum of 3 per month for conventional (**CON**) herds, and are dependent on the year (DFO, 2010). Some of the strategies used to increase production during incentive days without increasing herd size include nutritional strategies (such as the use of SFA supplements in the lactating cow ration) and extending the lactation of cows; both have the potential to increase FFA in milk (de Koning et al., 2003; Wiking et al., 2003). The number of incentive days and strategies to fill incentive days differ for organic and grass-fed herds. For example, fat supplementation is not permitted in organic or grass-fed herds (Pustjens et al., 2017).

The type of milking system can also influence FFA levels (Wiking et al., 2019; Woodhouse et al., 2025c). The literature consistently reports that nonparlor milking systems (such as tiestalls and automated milking systems [**AMS**]) are associated with higher FFA compared with parlor-milked herds (Wiking et al., 2019; Woodhouse et al., 2025c).

Although there has been research to identify some of the on-farm factors associated with the level of FFA, few studies have explored the impact of monthly and yearly factors, such as milk component changes and the offering of production incentive days, which are unique to the Canadian dairy industry. The objective of this study was to investigate potential associations between milk composition, farm milking system type, and industry-level production incentives with elevated bulk tank milk FFA using a subset of ON CON farms enrolled in milk recording. It was hypothesized that nonparlor herds, incentive days, increased milk fat, decreased milk protein, and increased BSN levels would be associated with elevated FFA in bulk tank milk.

All licensed ON CON dairy herds that were registered with the Lactanet DHI milk recording program between August 2018 and December 2022 were included in the study. Only herds from the province of ON were included because FFA was either not being measured or was not readily available in the other Canadian provinces. Non-CON farms (3% of ON farms) were excluded because they have different production incentive programs. Excluding non-milk-recorded herds was necessary because farm milking system data were only available through the Lactanet DHI milk recording program.

Bulk tank milk composition data for each pick-up from each farm between August 2018 to December 2022 were obtained from DFO. The composition data of interest collected and tested at every milk pick-up included FFA (mmol/100 g of fat), milk fat (% weight/volume), milk protein (% weight/volume), SCC (thousand cells/mL), and milk shipment yield (L). BactoScan (thousand bacteria/mL) levels were also recorded, but they were less frequent because levels were only tested once a week. All milk components were analyzed at the University of Guelph Food Laboratory (Guelph, ON, Canada). Free fatty acid concentrations were measured using infrared spectroscopy with MilkoScan machines by FOSS (Wiking et al., 2017).

Monthly average values for all components and yields were then generated for each farm to minimize the effect of day-to-day variation. Upon inspection, the average FFA values in February 2020 and April 2021 were markedly different from the other months, and it was suspected that a laboratory calibration error had occurred. Therefore, those months were removed from the dataset. Average FFA values for each monthly average observation were categorized as normal (<1.2 mmol/100 g of fat) or elevated (≥1.2 mmol/100 g of fat; Wiking et al., 2017). The number of incentive days (0–3) during each month was obtained from DFO and added to the dataset. Given that some herds changed milking systems during the study period, milking system type was identified by month for each farm using data from Lactanet DHI.

Data were analyzed using commercial statistical software (STATA version 16.0, StataCorp). Researchers and participants were not blinded. All continuous variables were assessed for linearity using locally weighted scatterplot smoothing. BactoScan results were not linear, so a geometric mean was used rather than an arithmetic mean. Milk volume was categorized by quartile based on average milk shipment volumes. The number of incentive days was categorized as 0, 1, 2, or 3 incentive days in the corresponding month of milk pick-up. Descriptive statistics were generated for all continuous variables and frequency distributions were reported for categorical variables.

A causal diagram was constructed to identify confounding and intervening variables. The average monthly milk fat percentage was identified as an intervening variable between incentive days and elevated FFA. This is because incentive days can increase milk fat percentage due to nutritional strategies used during incentive days, such as increased fat supplementation use in the lactating ration (Tagliapietra et al., 2007; Dos Santos Neto et al., 2021). Month and year were included in the mixed multivariable model to account for their effects because FFA and incentive days can change on a monthly and yearly basis (Woodhouse et al., 2025b). Herd size was identified as a confounding variable for milking system because national data suggest that tiestall systems and AMS have smaller herd sizes than parlor milking systems (Lactanet, 2022). No interactions were detected. None of the variables were highly correlated (r > |0.8|) based on an examination of Spearman's rank correlation coefficient.

A univariable and then multivariable mixed-effect logistic regression model were used. A logistic model was chosen because the outcome of interest (elevated monthly average FFA concentration) was dichotomous and there was a sufficient sample size of herds with elevated monthly average FFA concentrations. A mixed-effect model was chosen to account for multiple observations from each farm. Herd was included in the model as a random effect, and the fixed effects included milk protein, BSN, SCC, milk volume, month, year, number of incentive days, and milking system. Each explanatory variable was tested for significance in a univariable mixed-effect logistic regression analysis. Those variables that had *P* < 0.05 at the univariable level were offered to the multivariable mixed-effect logistic regression model. Backward selection was used until all variables in the mixed multivariable logistic regression model had *P* < 0.05, and the corresponding odds ratio (**OR**) and 95% CI were reported.

There were 3,009 ON CON farms with milking system type data between August 2018 and December 2022. Fifty-three percent of these farms had tiestall milking systems (n = 1,578), 29% had a parlor (n = 878), and 18% had AMS (n = 553). From these, 148,965 monthly FFA average observations were included in the analysis. The average monthly bulk tank FFA was 0.83 mmol/100 g of fat (SD = 0.32, range 0.04 to 7.68), and 7% (n = 10,016) of monthly averages were elevated (≥1.2 mmol/100 g of fat). The summary bulk tank milk characteristics and milk component data are presented in Table 1, Table 2.

**Table 1:** Descriptive statistics of monthly average (avg) milk components on Ontario conventional dairy farms registered with the Lactanet DHI milk recording program from August 2018 to December 2022

Variable	n	Mean	Median	SD	Minimum	Maximum
Avg monthly FFA (mmol/100 g of fat)	148,993	0.83	0.79	0.32	0.04	7.68
Avg monthly fat (% weighted volume)	148,965	4.16	4.13	0.30	2.83	6.25
Avg monthly protein (% weighted volume)	148,965	3.23	3.21	0.18	2.65	4.42
Avg monthly BSN (thousand bacteria/mL)	145,897	29.11	12	323.11	1.00	57,012
Log avg monthly BSN	145,897	2.62	2.48	0.82	0	10.95
Avg monthly SCC (thousand cells/mL)	148,965	192.16	181	80.25	18	820

**Table 2:** Frequency distributions of categorical variables included in the analysis of factors associated with monthly average (avg) elevated (≥1.2 mmol/100 g of fat) free fatty acid (FFA) concentration on Ontario conventional dairy farms registered with the Lactanet DHI milk recording program from August 2018 to December 2022

Variable	n	Percentage
Elevated FFA (≥1.2 mmol/100 g of fat)		
No	138,977	93.28
Yes	10,016	6.72
Year		
2018	14,413^1^	9.68^1^
2019	34,494	23.16
2020	32,302	21.68
2021	32,324	21.70
2022	35,432	23.79
Month		
January	11,649	7.82
February	8,719	5.85
March	11,634	7.81
April	8,714	5.85
May	11,655	7.82
June	11,690	7.85
July	11,741	7.88
August	14,630	9.82
September	14,637	9.83
October	14,633	9.82
November	14,631	9.82
December	14,632	9.82
Milking system		
Parlor	43,440	29.16
Tiestall	78,505	52.69
AMS	27,048	18.15
Milk volume quartiles (L)		
1 (476–35,984)	37,243	25.00
2 (35,985–53,712)	37,243	25.00
3 (53,713–86,620)	37,238	25.00
4 (86,621–1,577,249)	37,241	25.00
Number of incentive days		
0	66,659	44.75
1	44,125	29.62
2	11,751	7.89
3	26,430	17.74

1 Note that FFA testing commenced in August 2018.

In the mixed univariable logistic regression analyses, all variables were significant (*P* < 0.05) and, therefore, offered to the mixed multivariable logistic regression model. In the mixed multivariable logistic regression analysis (Table 3), all explanatory variables were significant except for SCC. The final model indicated there were greater odds of monthly average elevated FFA in months with 3 incentive days, lower milk protein levels, and higher BSN levels. Nonparlor milking systems, especially tiestalls, were also associated with higher odds of monthly average elevated FFA levels.

**Table 3:** Final mixed multivariable logistic regression model of significant factors associated with monthly average elevated (≥1.2 mmol/100 g of fat) free fatty acid (FFA) concentration on Ontario conventional dairy farms registered with the Lactanet milk recording program from August 2018 to December 2022^1^

Variable	OR	95% CI	*P*-value
Avg monthly protein (% weighted volume)	0.03	0.02, 0.04	<0.001*
Avg monthly BSN (×1,000 bacteria per mL)	2.95	2.80, 3.11	0.002*
Incentive days			
0	Referent		
1	1.07	0.97, 1.18	0.185
2	1.05	0.89, 1.24	0.586
3	1.58	1.31, 1.91	<0.001*
Milking system			
Parlor	Referent		
Tiestall	28.64	14.18, 57.90	<0.001*
AMS	10.94	5.19, 23.09	<0.001*
Milk volume quartile (L)			
1 (476–35,984)	Referent		
2 (35985–53,712)	0.39	0.34, 0.44	<0.001
3 (53713–86,620)	0.20	0.17, 0.25	<0.001
4 (86621–1,577,249)	0.10	0.07, 0.13	<0.001
Year			
2018	1.52	1.29, 1.79	<0.001
2019	1.01	0.90, 1.12	0.922
2020	1.17	1.05, 1.29	0.004
2021	Referent		
2022	1.48	1.34, 1.65	<0.001
Month			
January	2.72	2.25, 3.27	<0.001
February	1.50	1.22, 1.85	<0.001
March	1.36	1.12, 1.65	0.002
April	2.00	1.64, 2.44	<0.001
May	Referent		
June	1.86	1.54, 2.25	<0.001
July	4.13	3.45, 4.95	<0.001
August	2.97	2.47, 3.57	<0.001
September	3.07	2.45, 3.84	<0.001
October	2.79	2.24, 3.47	<0.001
November	2.65	2.11, 3.33	<0.001
December	2.61	2.16, 3.16	<0.001

1 Odds ratios (OR), 95% CI, and *P*-values are presented.

* Significant at *P* < 0.05.

The odds of elevated monthly average FFA were greater for tiestall farms (OR = 28.6, 95% CI [14.2, 57.9], *P* < 0.001) and AMS farms (OR = 10.9, 95% CI [5.2, 23.1], *P* < 0.001) when compared with parlor farms. This relationship was expected because it is well documented in the literature that parlor milking systems have average lower FFA levels (Klungel et al., 2000; Wiking et al., 2019; Woodhouse et al., 2025c). Both tiestall systems and AMS are associated with smaller herd sizes than parlor systems, and this is a risk factor for increased FFA levels due to individual cow FFA levels representing a larger proportion of the bulk tank (Wiking et al., 2019). In addition, a farm with too large a bulk tank for a smaller herd size can risk FFA due to milk freezing (Wiking et al., 2019; Woodhouse, 2024). Higher FFA levels in nonparlor herds may also be attributable to system design, where tiestall systems and AMS have narrower and longer milk pipelines with more elevated sections than parlor milking systems (Wiking et al., 2019; Woodhouse et al., 2025c). The pipeline layouts in tiestall systems and AMS require more air admission than parlor milking systems, which can increase induced lipolysis (Rasmussen et al., 2006; Wiking et al., 2019; Woodhouse et al., 2025c).

Tiestall farms had greater odds of monthly average elevated FFA than AMS farms (OR = 2.6, 95% CI [2.0, 3.5], *P* < 0.001). Researchers have previously identified tiestall systems to less commonly have precooling, which can increase the risk of elevated FFA levels due to milk freezing (Wiking et al., 2019; Woodhouse et al., 2025c). Many tiestall systems are older and have air leakages within the pipeline or milking unit (Suzuki et al., 2022), which could contribute to the higher odds of elevated FFA compared with AMS observed in this study. Automated milking system FFA risk factors such as increased milking frequency and reduced filter change frequency (Wiking et al., 2006, 2019; Woodhouse et al., 2025a) are more easily managed on the farm, which could further explain fewer AMS farms with elevated FFA levels. It is noteworthy that there are significantly fewer AMS herds on milk recording than herds with tiestall systems (Lactanet, 2022), and this could have potentially affected the results of this study.

When controlling for month and year in the model, higher odds of monthly average elevated FFA levels were detected during periods with 3 incentive days compared with none (OR = 1.6, 95% CI [1.3, 1.9], *P* < 0.001). However, there was no association between monthly average elevated FFA and 1 or 2 incentive days. This result may suggest that ON CON dairy producers are more likely to make herd management changes to fill the quota during periods with higher incentive days. Strategies to fill the quota include adding dietary fatty acid supplements to milking cow rations, such as palm fat. Palm fat is high in SFA, which may contribute to an increase in FFA levels due to the large and unstable milk fat globules that are prone to induce lipolysis (Wiking et al., 2003, 2005, 2019). Another strategy used to fill the quota during incentive days is to keep more cows in milk by extending the lactation of cows. However, late-lactation cows are more likely to produce high FFA milk due to increased milk lipoprotein lipase content that can result in spontaneous milk fat breakdown (Hickey et al., 2006).

Lower milk protein was associated with increased odds of elevated monthly average FFA (OR = 33.2 per weighted volume percentage of milk protein decrease, 95% CI [24.2, 45.7], *P* < 0.001). The magnitude of this relationship suggests that sufficient milk protein levels are crucial to avoiding an elevated monthly average FFA concentration. Protein levels can vary depending on factors such as ration ingredients, cow health, and breed. Hanuš et al. (2008) reported that FFA levels can rise when a cow is dealing with negative energy balance. Rations that are low in protein or energy can decrease propionic acid concentration in the rumen and increase fat mobilization, leading to FFA production (Hanuš et al., 2008). Genetics, including breed, can also influence milk protein levels, but research is conflicting (Woodhouse and Kelton, 2023). Protein also helps form the milk fat globule membrane, which assists in its stabilization and prevention of induced lipolysis (Jenkins and McGuire, 2006; Hofstetter et al., 2014). The relationship between lower milk protein levels and higher FFA has been documented in other studies (Thomson et al., 2005; Hanuš et al., 2008; Marcondes et al., 2014). Hanuš et al. (2008) detected an average FFA increase of 0.70 mmol/100 g of fat with a milk protein decrease of 1%.

In agreement with our hypothesis, BSN was associated with monthly average elevated FFA (OR = 3.0 per unit increase in BSN [1,000 bacteria per mL more], 95% CI [2.8, 3.1], *P* = 0.002). This supports the mechanism of elevated FFA through increased bacterial lipolytic activity due to higher bacterial counts. The association with elevated FFA may have been even stronger if only psychotropic bacteria (the main bacteria contributing to lipolysis) were included, as not all bacteria have lipolytic effects (Deeth, 2006). In addition, our BSN data were limited due to less frequent testing than the other milk components studied. Hanuš et al. (2008) previously detected an association between FFA levels and BSN and SCC; however, in our study, SCC was not significant at the multivariable level. Somatic cell count may have been significantly associated with monthly average elevated FFA at the multivariable analyses level if more late-lactation or high SCC herds were included (Hickey et al., 2006).

Elevated monthly average bulk tank FFA concentrations on ON CON dairy farms were associated with incentive days, lower milk protein levels, higher BSN counts, and milking system type. Seven percent of the monthly average FFA concentrations over the 2018–2022 years were elevated (≥1.2 mmol/100 g of fat), and many of these samples came from nonparlor milking systems, especially tiestalls, which have more FFA risk factors to be aware of. Months with 3 production incentive days had increased odds of monthly average elevated FFA levels, likely attributable to feed and herd management changes to fill the quota. Lower milk protein levels were greatly associated with elevated monthly average FFA. Higher bacterial counts in milk were associated with elevated monthly average FFA, emphasizing that hygienic strategies on the farm should be prioritized. This research suggests that milk component, farm, and industry-level factors should be considered when mitigating FFA, and future research should investigate the management factors contributing to each.
